# Citizen science charts two major “stomatotypes” in the oral microbiome of adolescents and reveals links with habits and drinking water composition

**DOI:** 10.1186/s40168-018-0592-3

**Published:** 2018-12-06

**Authors:** Jesse R. Willis, Pedro González-Torres, Alexandros A. Pittis, Luis A. Bejarano, Luca Cozzuto, Nuria Andreu-Somavilla, Miriam Alloza-Trabado, Antonia Valentín, Ewa Ksiezopolska, Carlos Company, Harris Onywera, Magda Montfort, Antonio Hermoso, Susana Iraola-Guzmán, Ester Saus, Annick Labeeuw, Carlo Carolis, Jochen Hecht, Julia Ponomarenko, Toni Gabaldón

**Affiliations:** 1 Centre for Genomic Regulation (CRG), The Barcelona Institute of Science and Technology, Dr. Aiguader 88, Barcelona, 08003 Spain; 2 Universitat Pompeu Fabra (UPF), Barcelona, 08003 Spain; 3 ISGlobal, Centre for Research in Environmental Epidemiology (CREAL), Barcelona, Spain; 4 Institute of Infectious Disease and Molecular Medicine (IDM), University of Cape Town (UCT), Anzio Road, Observatory, Cape Town, 7925 South Africa; 5 Institució Catalana de Recerca i Estudis Avançats (ICREA), Pg. Lluís Companys 23, 08010 Barcelona, Spain

**Keywords:** Oral microbiome, Metagenomics, Stomatotypes, Tap water composition

## Abstract

**Background:**

The oral cavity comprises a rich and diverse microbiome, which plays important roles in health and disease. Previous studies have mostly focused on adult populations or in very young children, whereas the adolescent oral microbiome remains poorly studied. Here, we used a citizen science approach and 16S profiling to assess the oral microbiome of 1500 adolescents around Spain and its relationships with lifestyle, diet, hygiene, and socioeconomic and environmental parameters.

**Results:**

Our results provide a detailed snapshot of the adolescent oral microbiome and how it varies with lifestyle and other factors. In addition to hygiene and dietary habits, we found that the composition of tap water was related to important changes in the abundance of several bacterial genera. This points to an important role of drinking water in shaping the oral microbiota, which has been so far poorly explored. Overall, the microbiome samples of our study can be clustered into two broad compositional patterns (stomatotypes), driven mostly by *Neisseria* and *Prevotella*, respectively. These patterns show striking similarities with those found in unrelated populations.

**Conclusions:**

We hypothesize that these stomatotypes represent two possible global optimal equilibria in the oral microbiome that reflect underlying constraints of the human oral niche. As such, they should be found across a variety of geographical regions, lifestyles, and ages.

**Electronic supplementary material:**

The online version of this article (10.1186/s40168-018-0592-3) contains supplementary material, which is available to authorized users.

## Background

The oral cavity is among the most heavily colonized areas of the human body and harbors the second most diverse human microbiome [1]. Previous studies of the oral microbiome have estimated the presence of around 10^8^ microbial cells per milliliter of saliva, and the presence of up to 700 distinct prokaryotic taxa, of which approximately one third cannot be cultured [2–4]. The mouth is also the site where the most prevalent human diseases occur, including caries, gingivitis, and periodontitis [1, 5, 6]. In addition, given the close connections of the oral cavity with the vascular system and the digestive and respiratory tracks, alterations of the mouth microbiota have been related with diseases that affect other body parts, such as diabetes or cardiovascular disease [1, 4]. Understanding the composition of the oral microbiome across individuals, and how it relates with lifestyle habits such as diet or hygiene, is important to achieve a proactive management of oral health. The analysis of the microbiome through the next-generation sequencing of 16S amplicons (i.e., 16S metabarcoding) offers a cost-effective approach to assess the overall composition of an individual’s microbiome [7, 8]. Previous studies have assessed the oral microbiome in relation with factors such as biogeography, environment, age, or ethnicity [7, 9, 10], or have focused on the effect of smoking [11, 12], diet [13–16], or hygiene habits [17–19]. On the more clinical side, some studies have uncovered alterations of the oral microbiota in prevalent diseases of the oral cavity including periodontal disease [20] and caries [3, 21]. In addition, previous studies suggest that intrinsic physiological parameters of the host such as enzymatic content of saliva relate to variations in the microbiome [22]. Although the mouth comprises several distinct niches, previous large-scale studies have mostly probed microbial composition of saliva. This fluid can gather bacteria and metabolites that originate from other oral niches, and appear to be representative of the overall oral microbiome [23]. Furthermore, considering that saliva tests offer an ideal non-invasive source for diagnosis, relationships of its microbial composition with the presence of several diseases such as cancer have been investigated [24–26]. Most previous studies have focused on adults, or very young infants, with studies on adolescents lagging behind. The largest dataset on adolescents so far corresponds to a longitudinal study of 107 individuals, including 27 monozygotic and 18 di-zygotic twin pairs [27]. This study suggested that environment is the main determinant of the oral microbiome with differences between mono- or di-zygotic twins not being significant. Here, we used a citizen science approach and 16S metabarcoding to assess the composition of the microbiome of the oral cavity among teenagers in Spain. We studied its variation with more than 50 parameters including geographical location, gender, and urban environment, as well as several dietary and hygiene habits. Our study showcases the use of a citizen science approach to generate hypotheses that can be further validated in subsequent studies.

## Results

### Data collection and analysis

One thousand five hundred fifty-five samples were collected from students (ages 13–15) and their teachers in 40 schools around Spain during Spring 2015 [see Additional file 1]. Sample collection was coupled to science communication activities aiming to raise awareness about the role of the microbiome in health and disease, the potential of sequencing and bioinformatics technologies, and the scientific career (see http://www.sacalalengua.org). Donors were asked to answer a questionnaire, including 54 questions [see Additional file 2], some of which proposed by citizens, about their health, and their dietary and hygiene habits. The pH of the donor’s saliva was measured prior to sample collection. Samples were obtained using oral rinse, from which cells were collected and frozen (see “Online methods”). DNA extracted from the samples were subjected to 16S profiling of the V3–V4 regions, using Illumina MiSeq technology, and processed bioinformatically (see Materials and methods). Data from 1319 samples that passed all the quality filters were explored in terms of the relationships of the microbiome composition, the questionnaire results, and other metadata (see Materials and methods).

### Oral microbiome diversity is structured into two major stomatotypes

**Fig. 1:**
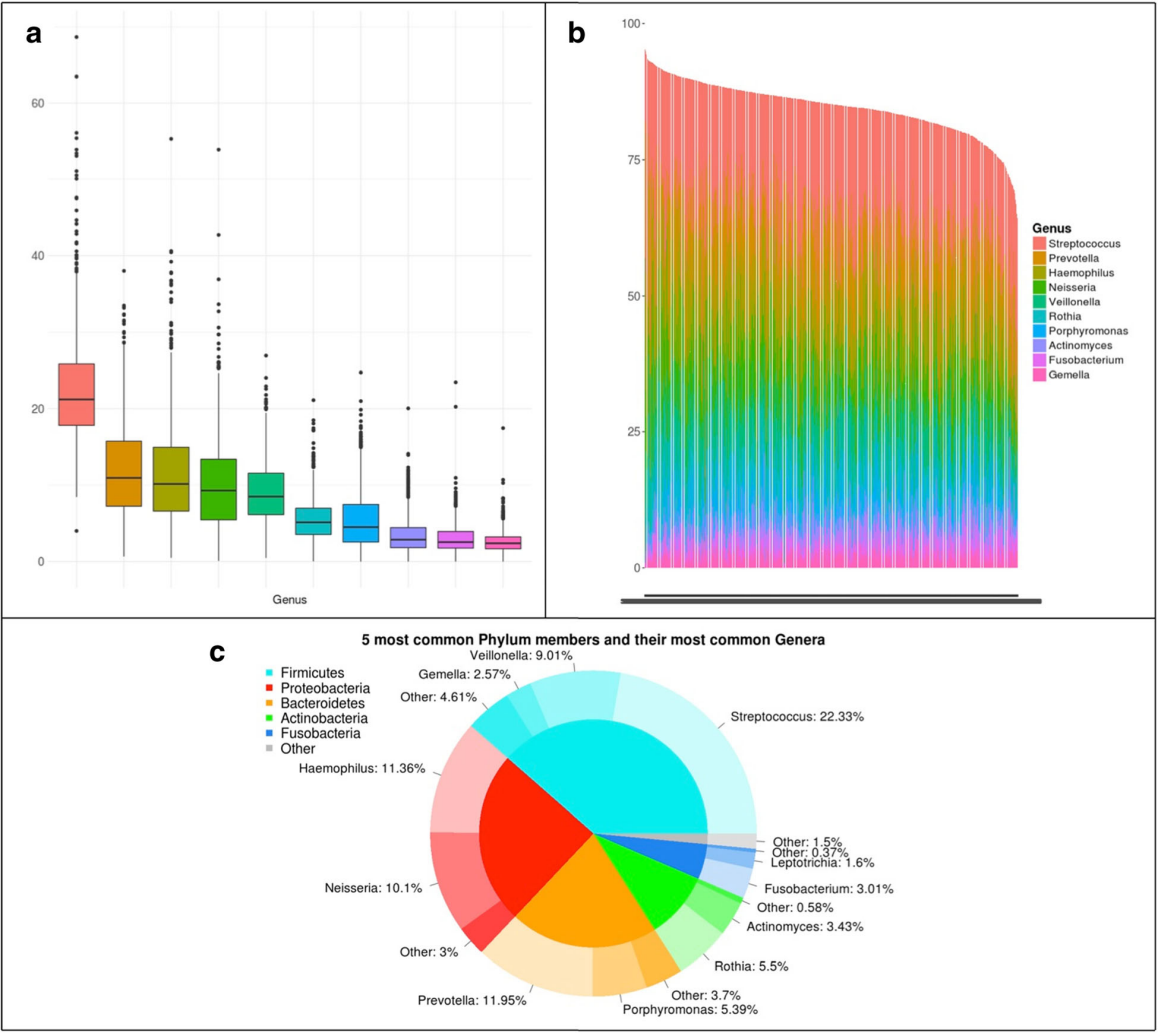
Microbiome composition. **a** Box plots of the relative abundances of the ten most common genera. **b** Stacked bars of relative abundances of the ten most common genera for all samples, showing the relative proportion of all samples made up of these ten genera. Stacked white bars are meaningless and appear due to lack of image resolution. **c** Donut chart showing the five most common phyla (inner ring) and the most common genera (outer ring) within each phylum with the average relative abundance per sample

**Fig. 2:**
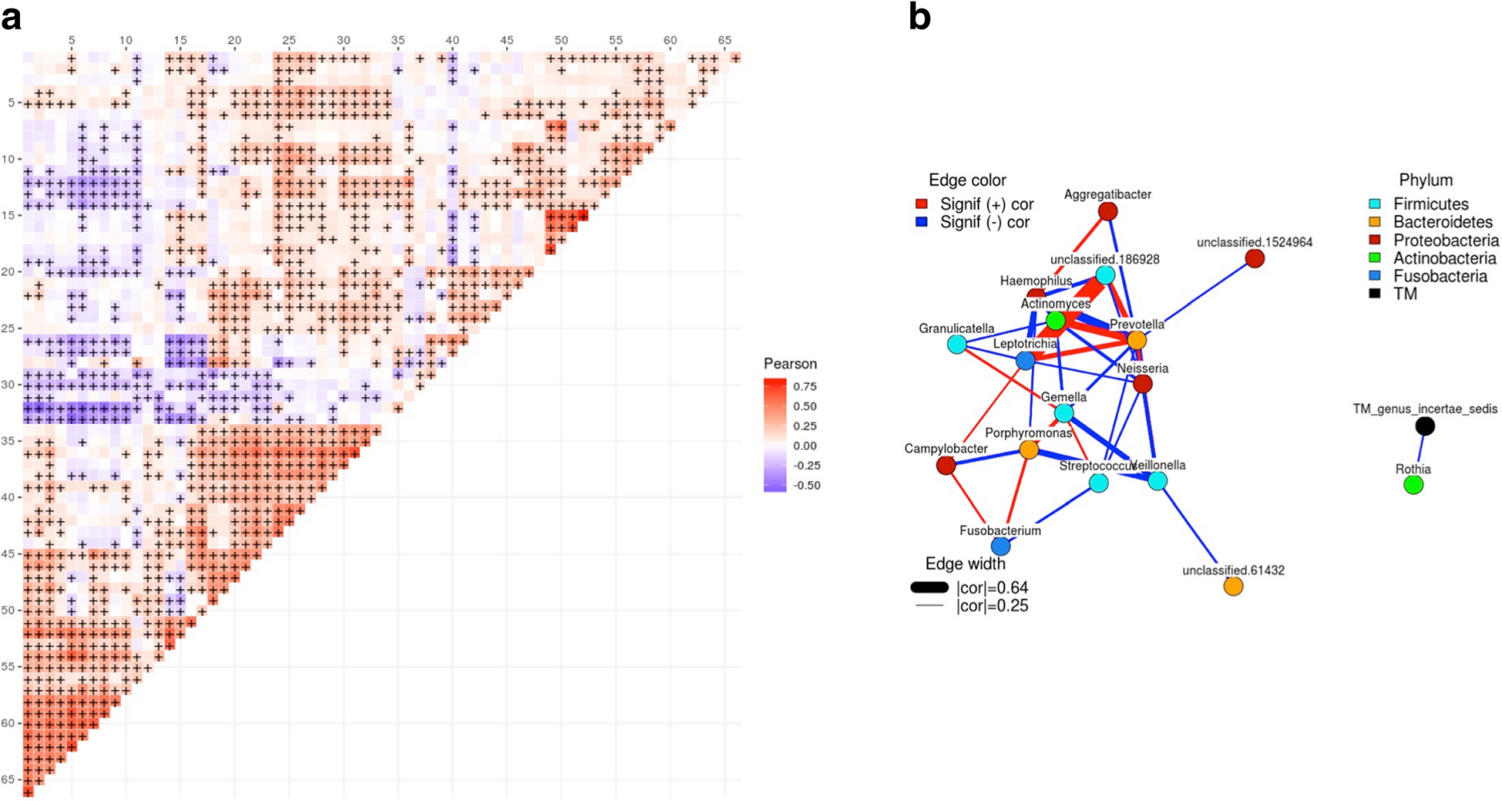
Correlations among genera (all samples). **a** Heatmap of correlations between relative abundances of genera. Color indicates Pearson correlation coefficient and “+” indicates a statistically significant correlation. While 332 different genera in total were detected, for the sake of visual representation, this figure shows only the 67 genera which were present in at least 1/3 of all samples (436). Correlation coefficient values for significant correlations can be found in Additional file 4. The indexes of genera within Additional file 4 are marked at every fifth position in the figure here so that names can be matched to the figure if so desired. **b** Co-occurrence network of the 20 most common genera. Edges indicate significant positive (red) or negative (blue) correlations between indicated genera. Edge width is proportional to Pearson correlation coefficient. Only displaying edges for coefficients of 0.25 or greater and − 0.25 or lower. The largest and smallest edge widths are shown with the corresponding absolute value of the correlation coefficient as it appears in the figure

**Fig. 3:**
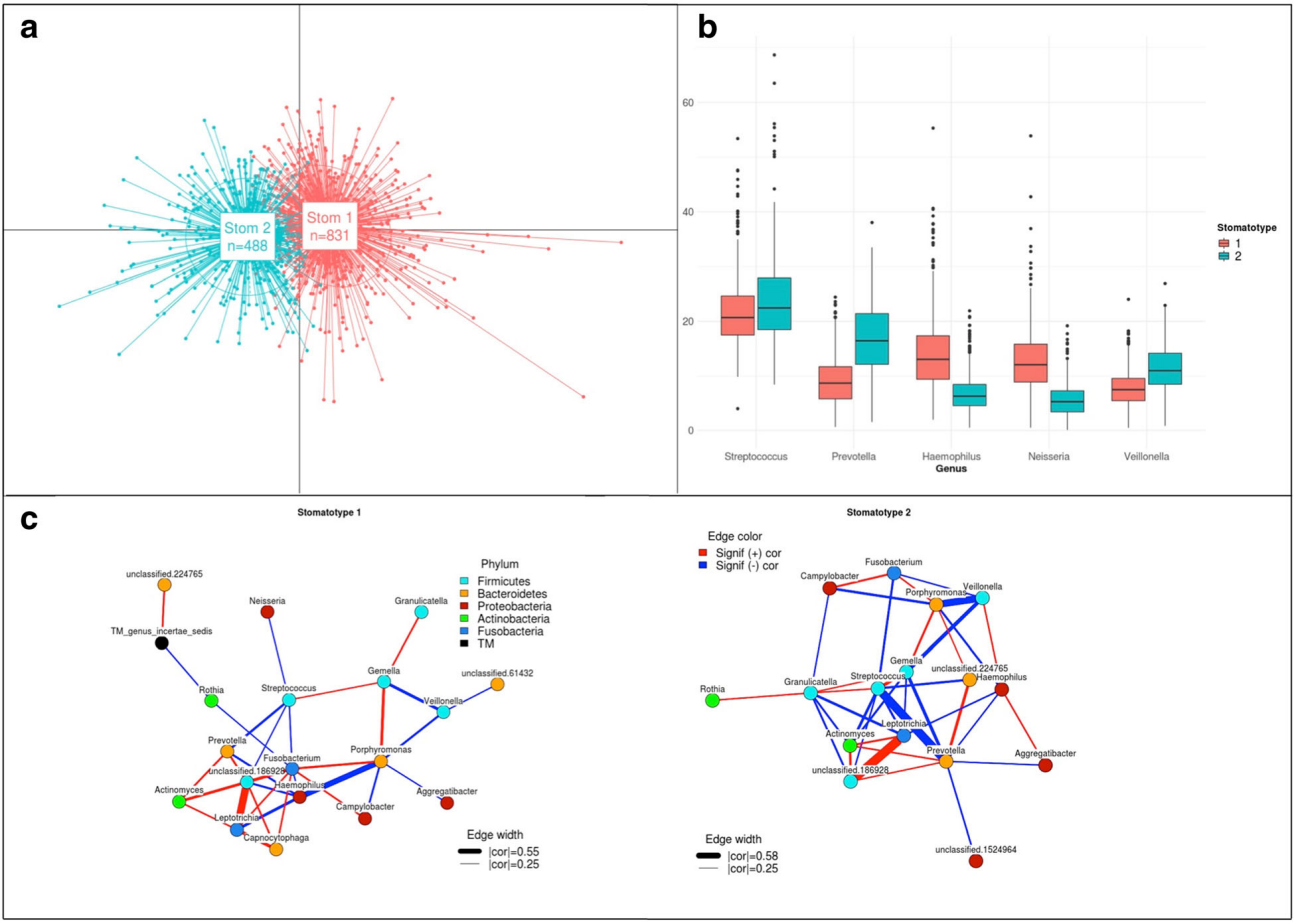
Stomatotypes. **a** Principal Coordinates Analysis (PCoA) of samples using a Jensen-Shannon Divergence (JSD). Shows that the samples cluster into 2 groups (stomatotypes). **b** Boxplots of relative abundances of the five most common genera in samples with stomatotype 1 (red) and stomatotype 2 (blue). Bonferroni-adjusted *p* values from Wilcoxon tests between samples of stomatotypes 1 and 2 for streptococcus is 1.1e−7, while the values for the other 4 genera here were all less than 2e−16. **c** Co-occurence networks of 20 most common genera within samples of Stomatotypes 1 and 2 separately. Edges indicate significant positive (red) or negative (blue) correlations between indicated genera. Edge width is proportional to Pearson correlation coefficient. Only displaying edges for coefficients of 0.25 or greater. The largest and smallest edge widths are shown with the corresponding absolute value of the correlation coefficient as it appears in the figure

**Fig. 4:**
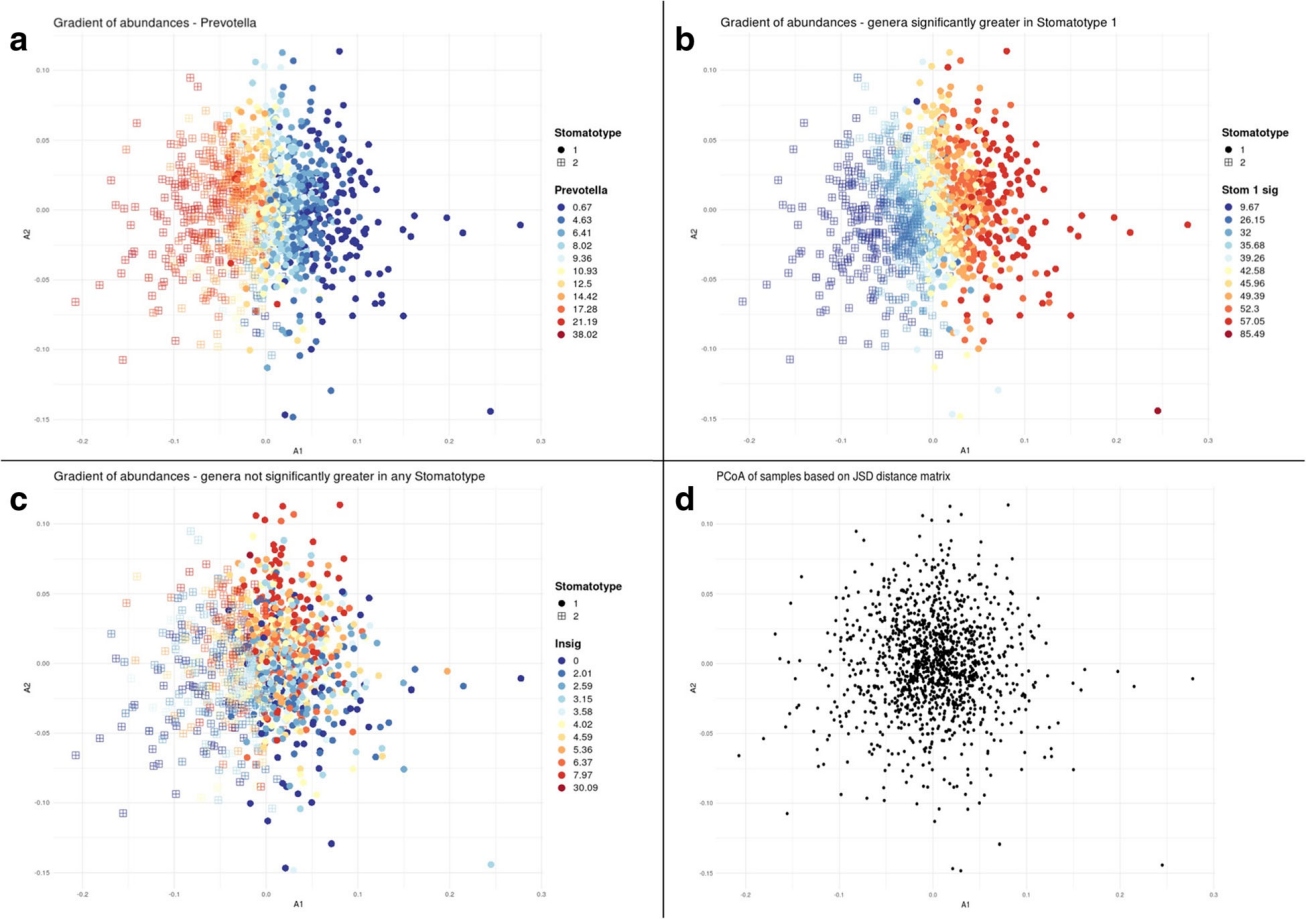
Gradients of abundances. Principal Coordinates Analysis (PCoA) of samples using a Jensen-Shannon Divergence (JSD) shows points with the same coordinates as in Fig. 3a. Circles indicate samples of stomatotype 1 and squares indicate samples of stomatotype 2. Colors represent abundance of the indicated genus (or the sum of abundances of indicated genera) for a given sample, where red is higher and blue is lower, with values indicated in the legend to the right. **a** Abundances for the genus *Prevotella*. **b** Sum of abundances of the 19 genera that were found to have significantly higher abundances in samples of stomatotype 1. **c** Sum of abundances of the 275 genera that did not have significantly higher abundances in samples of either stomatotype over the other. **d** Points without color or shape in order to display the spread of samples within the PCoA

Our analyses provide a snapshot of the microbial diversity in oral samples in young adolescents across Spain, and do so with unprecedented scale and resolution (Fig. 1). Overall, we identified 332 operational taxonomic units (OTUs) at the genus level in our dataset. Thirty-two genera were common, appearing in 75% or more of the sampled individuals. This “core” set comprised typical oral bacteria. The top ten most abundant genera represented collectively 84.64% of the analyzed sequences and were present in 99.6% of the samples. *Streptococcus* was the most abundant genus in most (68%) samples and showed an average relative abundance of 22.3%, followed by *Prevotella* (11.9%), *Haemophilus* (11.4%), *Neisseria* (10.1%), and *Veillonella* (9%). This core community composition and distribution is consistent with previous studies of oral healthy microbiomes [2, 9, 22, 28–30]. For instance, 20 of our 32 common genera are also common in a recent study of the oral microbiome of 2343 adults in Hisayama (Japan) [9]. Similar to previous oral microbiome surveys [7, 29], we found high alpha (within sample) diversity (mean Shannon diversity 2.5, see Additional file 3) and low beta (between samples) diversity (mean weighted UniFrac distance 0.118). Overall correlations among taxa across all samples revealed several clusters of co-occurring genera that hint to underlying ecological interactions (Fig. 2, Additional file 4). For instance, strong co-occurrence links *Leptotrichia*, *Actinomyces*, and *Prevotella*, and this latter one with *Veillonella*, suggesting they may be ecologically related. Genera in this cluster tend to anti-correlate with *Haemophilus*, *Porphyromonas*, and *Gemella*. Previous studies have shown that individual microbiomes from certain niches can be clustered into different types, such as the enterotypes of the gut microbiome [31]. Using this approach on our data (see Materials and methods) results in two major clusters, which we here refer to as “stomatotypes” in analogy to the enterotypes of the gut microbiome. The two defined stomatotypes differ in their microbial composition and abundance covariations, and for which *Neisseria* (stomatotype 1) and *Prevotella* (stomatotype 2) are the genera driving most differences (Fig. 3). Other differences include higher proportions of *Haemophilus* in stomatotype 1 and higher proportions of *Veillonella* and *Streptococcus* in stomatotype 2. Importantly, although the studies are performed with different methodologies and have largely different target populations, we noted a strong parallelism between our two stomatotypes and the defined “coinhabiting groups” in the abovementioned Hisayama study [9]. Of note, other studies of the oral microbiome have found a different number of clusters. An analysis of the oral microbiome in 268 healthy young adults (18–32) classified the samples into five discrete clusters [22], whereas another study of 161 healthy adults found three different clusters [16]. Yet, many parallels can also be found between our stomatotypes, and those in these studies. In terms of the driving species, our stomatotypes 1 and 2 are similar, respectively, to MIC1.3 and MIC2 of the 268 adults study and to clusters 1 and 2 of the 161 adults study. These striking similarities between disparate studies suggest that these two major stomatotypes may be ubiquitous and define global equilibria in the human mouth microbiome. As we discuss below, these stomatotypes are not discrete, well separated entities, but rather represent two poles of a gradient of microbial compositions. The stomatotypes are driven mostly by certain abundant genera, but do not explain the variability found in many other genera. This is apparent when plotting the abundance of different genera onto the principal coordinate analyses (see Fig. 4).

**Fig. 5:**
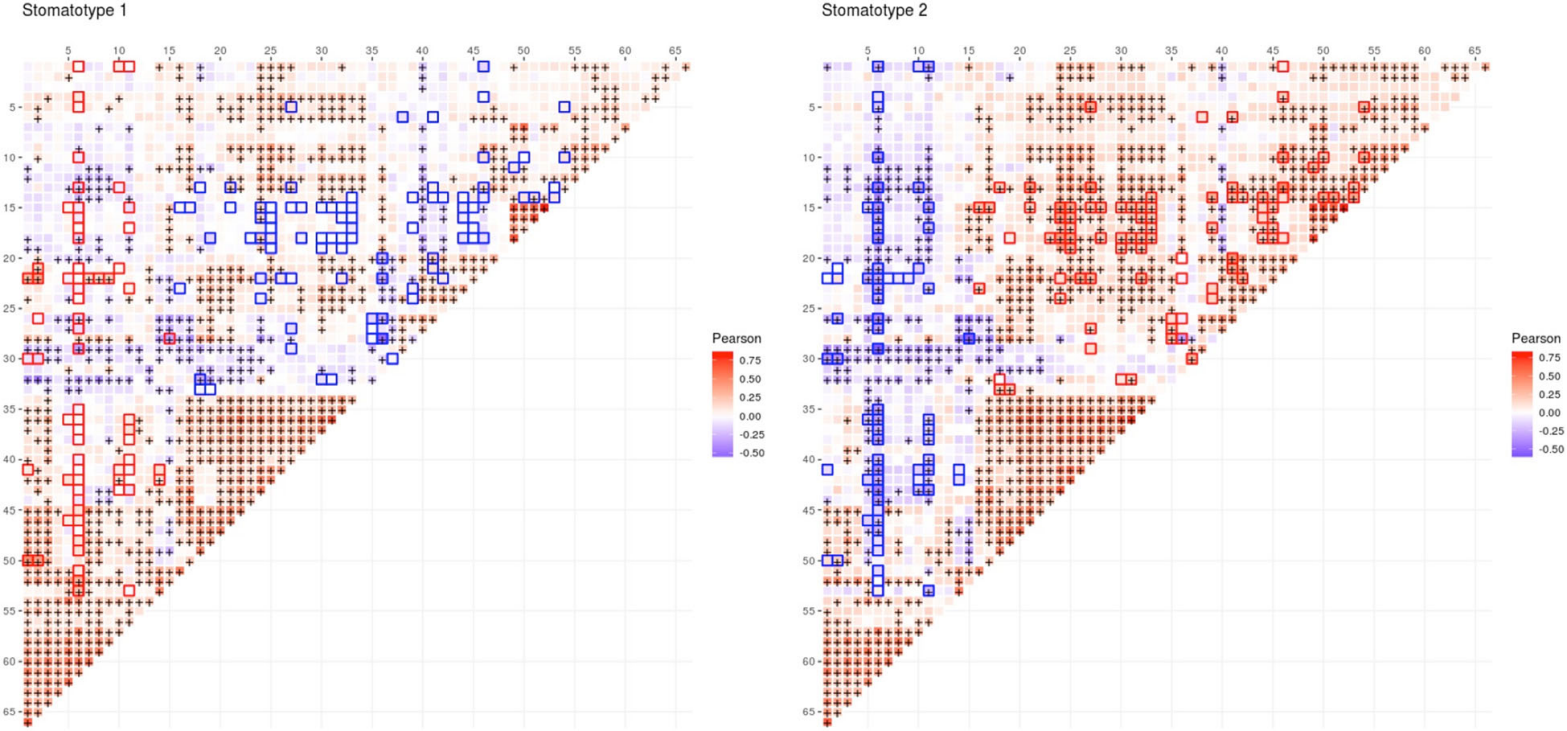
Correlations among stomatotypes. Heatmap of correlations between relative abundances of genera in samples with stomatotype 1 (left) and stomatotype 2 (right). Color indicates Pearson correlation coefficient and “+” indicates a statistically significant correlation. Highlighted boxes indicate genera pairs for which the correlation coefficient in the given stomatotype is at least 0.2 greater (red) or lower (blue) than the correlation coefficient in the other stomatotype. While 332 different genera in total were detected, for the sake of visual representation, this figure, as in Fig. 3, shows only the 67 genera which were present in at least 1/3 of all samples (436). Row and columns are ordered as in Fig. 2 and thus can also be compared with Additional file 4 in the same manner mentioned in Fig. 2

Studying co-occurrence patterns for each stomatotype separately reveals underlying bacterial communities that are shared or specific (Fig. 5). In both stomatotypes, *Streptococcus* is positively correlated with *Gemella* and negatively with *Prevotella and Fusobacterium* (Fig. 3c). This observation fits with recent studies describing oral plaque formation and evolution in disbiotic processes that have led to the formulation of the “ecological plaque hypothesis” [1, 32–34]. In this model, *Streptococcus*, *Gemella*, and *Neisseria* are among the pioneer colonizers that contribute to initial plaque formation. These genera are replaced in further evolution of the plaque by anaerobic species of several genera, including *Prevotella*, *Porphyromonas*, *Fusobacterum*, and *Veillonella*. Thus, the abundance covariations observed in both stomatotypes may partly reflect the underlying diversity of biofilm succession stages comprised in our samples and would support the main axis of previously observed core community changes in dental plaque. Positive correlations between *Porphyromonas* and *Fusobacterium* and negative correlations between *Veillonella* and *Gemella* further support this model, while positive correlations between *Porphyromonas* and *Gemella* and negative correlations between *Porphyromonas* and *Veillonella* would not be explained by the current plaque succession model. Of note, several of the correlations mentioned in our study coincide with those found in previous studies [16, 22]. Microbial compositions in oral rinse samples can only be considered a proxy for plaque communities, as the procedure collects cells from different oral niches. However, earlier studies using similar collection protocols and including information on plaque status or dental health have found correlations between microbial composition of saliva, the amount of plaque and diseases such as periodontitis, or caries [5, 9, 35, 36].

Although many of the covariations between the two stomatotypes are similar, their strengths can be markedly different. In addition, some covariations appear specific for each stomatotype. For instance, in the case of stomatotype 1, we detected positive covariation of *Fusobacterium* and *Capnocytophaga*, both anaerobic bacteria implicated in dental plaque progression [33], while in stomatotype 2, we specifically detect antagonism between *Streptococcus* and *Actinomyces*, which are known to compete in the initial phases of dental plaque formation [32, 37]. Thus, the two stomatotypes may point to differences in the relative impact of underlying processes and microbial communities that differentially affect individuals in our study.

### Lifestyle and social parameters

We next explored correlations between social parameters, questionnaire answers, and microbial composition [see Additional files 5, 6, 7, and 8]. We found that living in rural or urban areas did not correlate with significant changes in the microbiome. This suggests that diets and lifestyles of students are similar in cities and rural areas in Spain, as confirmed by our questionnaire, which only revealed significant differences in terms of a higher likelihood of having dogs for students living in the country side. Socioeconomic status did correlate significantly with the abundance of some genera, positively with *Rhizobium* and negatively with *Bradyrhizobium*, *Acinetobacter*, and *Pseudomonas.* Here, some differences in dietary habits were found, with a lower socioeconomic status being correlated with higher consumption of coke and sweets among students. We found no large differences between oral microbiomes of males and females, with only two genera (*Actinomyces* and *Oribacterium*) showing significantly different abundances (both higher in males). Boys and girls had some different habits. While the former tended to drink more milk, coke, or energetic drinks, the latter chewed gum and brushed their teeth more often. Larger differences in the oral microbiome were found between students and their teachers. Teachers’ microbiomes were enriched in *Alloscardovia*, *Parascardovia*, *Filifactor*, *Bulleidia*, *Mycoplasma*, *Phocaeicola*, *Hallella*, *Howardella*, *Anaeroglobus*, *Dialister*, *Desulfobulbus*, and *Campylobacter*, while those of students were enriched in *Actinomyces*, *Abiotrophia*, *Granulicatella*, *Rhizobium*, *Burkholderia*, and *Ralstonia*, with the latter two genera being absent from any of the teacher’s samples. These large differences may be related to age but also to their understandably different lifestyle. The students were more often consuming sweets and chewing gum, while teachers were consuming significantly more coffee and alcohol, reported more dental health problems, and used flossing more frequently. Although not the focus of the study, some interesting correlations did emerge among the items in the questionnaire. For instance, smokers tend to consume more alcohol, and students who reported having a kissing partner were more likely to smoke, drink alcohol, or chew gum [see Additional file 6]. Interestingly, students with kissing partners had a higher number of taxa in their microbiomes, which also showed a significantly higher presence of *Treponema.* Importantly, the reported consumption of alcohol among 314 students was associated with a higher presence of several bacterial genera including *Mycoplasma*, *Filifactor*, *Treponema*, and *Desulfobulbus*, among others [see Additional file 7]. Although 108 students declared smoking occasionally, we did not detect significant differences in their microbiomes. *Gemella* negatively correlated with the consumption of yogurt and milk. In addition, the consumption of milk was positively correlated with the of *Actinomyces* and *Atopobium*.

### Hygiene habits and saliva pH

Acidification plays an important role in oral health problems such as caries or periodontitis [3, 38]. A pH level of less than 5.5 can put a person at risk of tooth enamel erosion, leading to the formation of cavities, while higher pH can reduce this risk. Measured oral pH in our samples had a median of 7.5 but showed a wide range [see Additional file 9]. Higher oral pH was positively correlated with the abundance of *Fusobacterium* and *Porphyromonas*, a bacterial genus known to grow optimally in alkaline environments, and able to increase the pH of its medium [38]. Other genera such as *Streptococcus* or *Veillonella*, among others, correlated negatively with saliva pH [see Additional file 7]. *Veillonella* species are known to increase their abundance in acidic environments derived from fermentation processes, such as those occurring in mature dental plaque [3]. Importantly, no hygiene or dietary habit was shown to impact saliva pH in our study [see Additional file 8]. Admittedly, measurements of saliva pH using pH strips—a limitation imposed in part by our citizen science approach—lack the precision provided by a pH meter (see “Materials and methods” section). However, all our detected correlations were robust to stochastic variations within the precision range of the measurement, as shown by 1000 randomization tests (see “Materials and methods” section).

Our questionnaire included several questions on oral hygiene and dental devices. Hygiene habits usually showed high correlations among themselves, so that people who brush their teeth more often tended to use fluoride supplements and floss and were more likely to wash their hands before eating and/or after using the bathroom. Additionally, people using braces were more often brushing their teeth, and those reporting past nerve extractions drank more alcohol. According to our data, differences in type and frequency of oral hygiene do have measurable effects in the oral microbiome. Frequency of brushing teeth correlated negatively with the relative abundance of *Gemella*, *Streptobacillus*, *Granulicatella*, and *Porphyromonas*. It is known that caries is generally associated with an increase of *Streptococcus*, but also of *Granulicatella*, and *Gemella* [21]—although in the latter case, this varies with age [39]—supporting the effect of brushing against primary dental plaque. In contrast, flossing or using supplemental fluoride mouth wash did not seem to significantly impact the oral microbiome*.* The presence of dental implants did not show any correlation with oral microbiome changes, but wearing orthodontic braces did correlate positively with the abundance of many genera. These included several anaerobic or facultatively anaerobic genera such as *Corynebacterium*, *Bifidobacterium*, *Parascardovia*, *Olsenella*, *Capnocytophaga*, *Lactobacillus*, *Dialister*, *Schwartzia*, *Selenomonas*, and *Cardiobacterium.* This suggests that such orthodontic devices and their surfaces may promote the proliferation of specific biofilm communities. Most of these genera comprise anaerobic Gram negative species or Gram positives associated to acidic fermentations, which are generally associated to mature biofilm acidification, as well as caries and periodontal disease [3]. *Selenomonas* has been described as one of the most abundant taxa during orthodontic braces treatment and has been linked to common oral diseases such as gingivitis [19].

### Tap water influences the oral microbiome

**Fig. 6:**
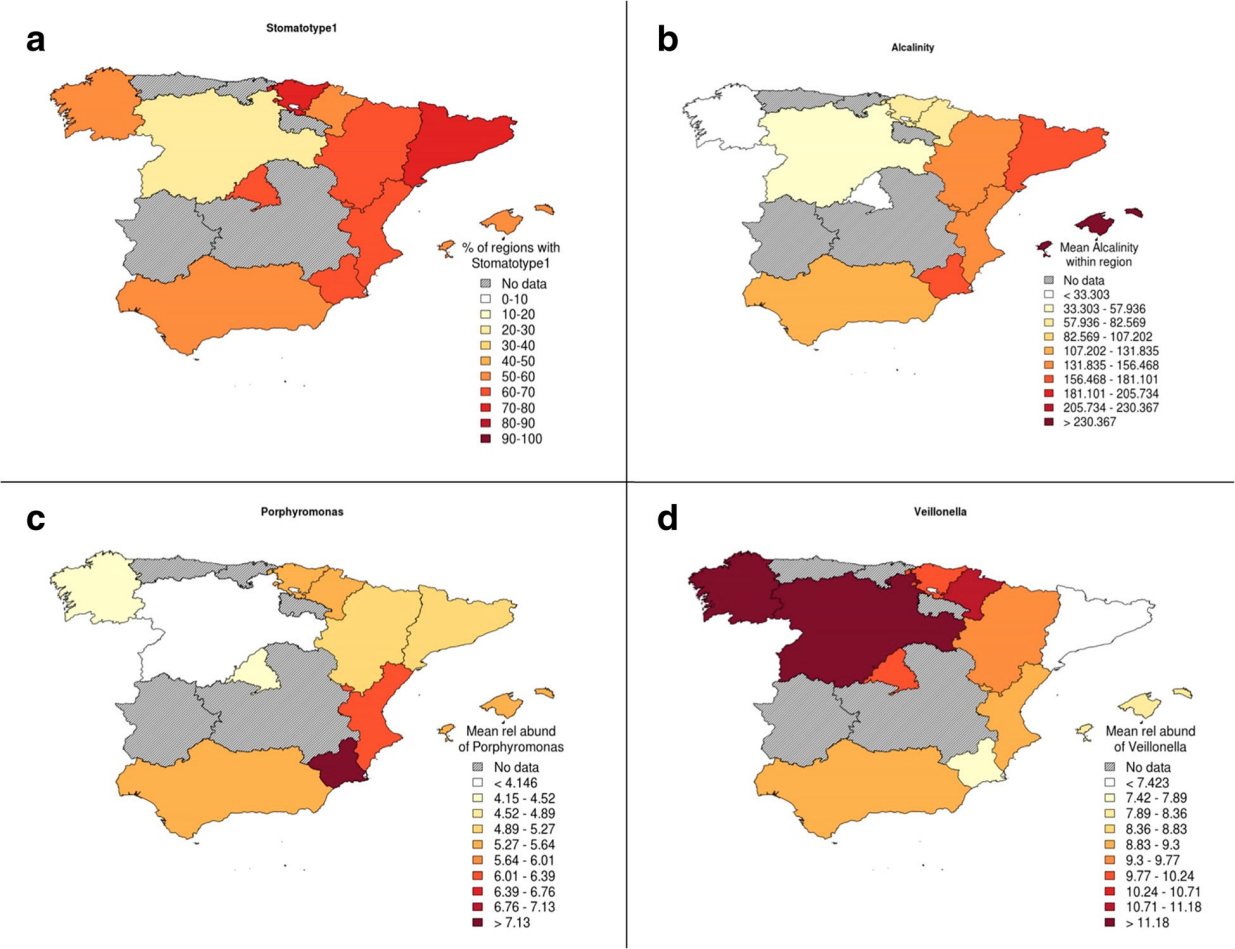
Geographical patterns. Maps show that most of the measured ion levels follow a similar pattern to the proportion of stomatotype 1 samples. *Porphyromonas* had a significantly higher abundance in stomatotype 1 samples, while *Veillonella* had a significantly higher abundance in stomatotype 2 samples. Region names can be seen in Additional file 1. **a** Percentage of samples from each region that have stomatotype 1. **b** Mean alkalinity level per sample in each region (an example of one of the tap water measurements compared in this study). **c** Mean abundance of *Porphyromonas* per sample in each region. **d** Mean abundance of *Veillonella* per sample in each region

**Fig. 7:**
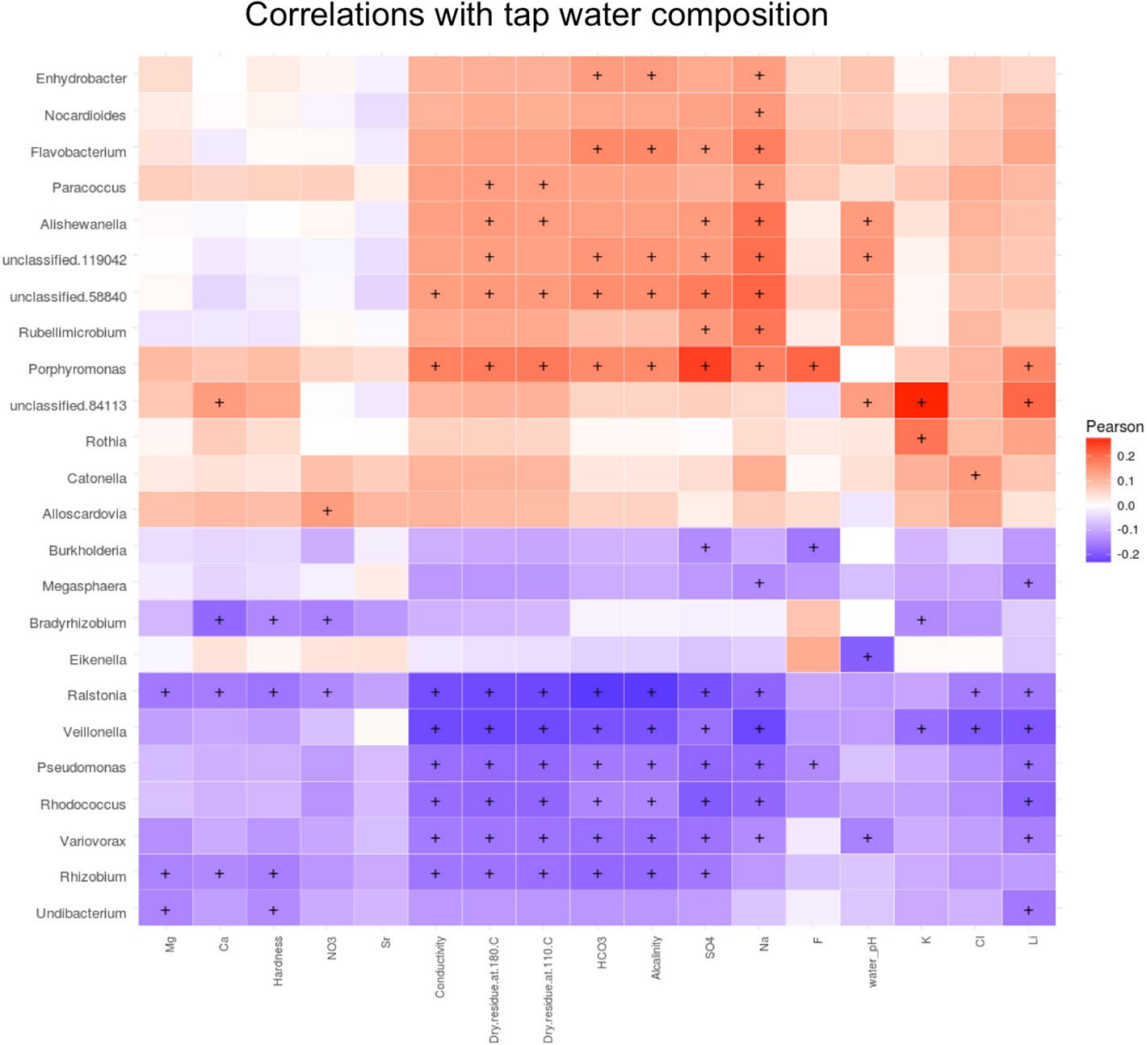
Correlations with tap water composition. Heatmap of correlations between relative abundances of genera with measurements of various components of tap water. Samples that primarily drank from bottled water (505 out of 1319) at home were excluded here. Color indicates Pearson correlation coefficients and “+” indicates a statistically significant correlation

Unexpectedly, we found no significant differences between lifestyle of students with the two oral stomatotypes, suggesting our data have not sufficiently captured the key factors underlying these different microbial communities. Notably, however, the two stomatotypes, and some genera, were geographically widespread but showed distinct abundance patterns, which suggest some environmental influence. The patterns were sometimes reminiscent of maps of certain public water quality parameters, such as alkalinity or water hardness, which differ significantly across regions in Spain (Fig. 6). In addition, the mouth is constantly exposed to tap water, which is consumed for drinking, cooking, and hygiene. Hence, we decided to investigate this factor in more detail and linked our samples to the chemical composition of tap water of the nearest town, as reported in recent studies [40–42]. For this analysis, we removed individuals that declared drinking bottled water. No strong correlation was found between the two stomatotypes and any of the 17 water parameters investigated. However, we found that most considered water quality parameters are associated to alterations in the composition of several genera (Fig. 7, Additional file 10). *Porphyromonas* was positively associated with the presence of fluoride (F) and sulfate (SO4) in tap water. A group of genera including, among others, *Veillonella*, *Ralstonia*, *Rhizobium*, *Rhodococcus*, and *Pseudomonas* negatively correlated with several of the following parameters: water hardness, alkalinity, conductivity, and the presence of SO_4_, magnesium (Mg), sodium (Na), calcium (Ca), chloride (Cl), and the amount of dry matter after boiling. Other genera correlated positively with several of these same variables, including *Porphyromonas* and *Flavobacterium*. *Ralstonia* abundance was also negatively affected by nearly all other water variables, and it was the genus whose abundance changed the most with tap water quality, followed by *Rhizobium*, *Veillonella*, and *Pseudomonas*. These results suggest that tap water composition may be an important, poorly studied factor shaping the oral microbiome.

## Discussion

Our study provides a comprehensive survey of the oral microbiome in Spanish adolescents, a target group that remains poorly explored. The citizen science approach has allowed us to address questions raised by citizens, train them in the use and interpretation of the data, and open a dialog with society on technologies and scientific questions of growing relevance. Although a citizen-based approach faces important limitations as compared to clinical studies, such as the difficulty to comprehensively evaluate clinical parameters by experts, it enables access to a large number of samples and of a different kind of those usually targeted by other studies. The high number of samples, the narrow range of geographical areas and ages under study, and the richness of collected metadata provide us an unprecedented level of resolution to study the adolescent oral microbiome. The insights gained from our study have served to generate working hypotheses regarding the composition and variability of the oral microbiome of adolescents that can be tested in future, more conventional studies. The core microbiome comprised typical oral bacteria that are commonly identified as abundant in similar oral microbiome surveys [28]. All genera discussed in the paper with the exception of *Rubellimicrobium* and *Undibacterium* have been previously identified in oral samples. Although the issue of contamination is a common theme in microbiome analyses, 20 amplification cycles and cell-rich starting materials such as oral samples are predicted to be minimally affected [43]. In accordance with this, all of our negative controls provided no measurable results and a negligible number of reads when forced into library preparation and sequencing (see the “Materials and samples” section). However, we cannot discard the possibility that some of the low abundance genera identified are not stable components of the oral cavity but result from sporadic colonization from the close environment of the donor (i.e., food, air, or water).

Overall, we see that the oral microbiome of Spanish adolescents is impacted by dietary, hygiene, and other lifestyle habits. Differences observed point to a differential impact of habits on the oral microbiome of adolescents. For instance, frequent teeth brushing was shown to affect the relative proportion of oral genera more than flossing, or the use of fluoride supplements.

Similarly, consumption of alcohol among adolescents seemed to impact the oral microbiome more than smoking. In contrast, we did not find many differences between genders or rural versus urban environment. Interestingly, some variables such as body mass index, which is generally associated to alterations in the gut microbiome, and it has been associated to changes in the oral microbiome in adults [44], seemed to have a minor impact on the mouth microbiome of adolescents in our sample. Some of these differences may relate to the fact that some habits, such as smoking or some dietary habits, may have just been recently established, or the habit is more sporadic in adolescents, and the effects in the microbiome will only be apparent after a prolonged period of sustained habit. In addition, the oral microbiome of adolescents may have specificities as a transition phase from childhood to adulthood. Adolescence is a stage with major hormonal and habit changes, which likely impact the oral microbial community. In fact, this period of life is associated with a sharp increase in the incidence and severity of gingivitis [45], which may be related to underlying oral microbiome changes. This highlights the importance of increasing our knowledge of the adolescent oral microbiome, as well as to undertake longitudinal studies over adolescent to adulthood phases of life. Altogether, the chemical composition of tap water was found to be the investigated factor with the highest impact on the composition of the oral microbiome. Although the presence of the most abundant genera of the oral microbiome such as *Streptococcus*, *Prevotella*, or *Haemophilus* (the top three in our samples) were not significantly affected by tap water, some genera among the ten most abundant were affected, including *Veillonella*, *Porphyromonas*, *and Gemella.* Our results thus raise the question of the role of drinking water in shaping the oral microbiome, suggesting a potentially important role. Previous studies have analyzed the relationship between the presence of fluoride and the incidence of caries [46], but the overall impact on the human oral microbiota of this and other factors remain unexplored. In this regard, experiments in mice have shown that the composition of tap water can be related with changes in the gut microbiome [47] and have an incidence in the progression of diseases such as diabetes [48]. Further research is needed to follow up the potential role of tap water in shaping the human oral microbiome.

We found that the oral microbiome of the studied population can be broadly classified into two different stomatotypes. Although the time since last tooth brushing was not controlled in our study, we do not think this would drive overall observed differences regarding stomatotypes as all students in one class were sampled at the same time and we found that differences in stomatotypes were not driven by school class. Importantly, our two defined stomatotypes show notable overlap with the two “coinhabiting” groups of bacteria identified in another large study [9]. Considering that the two studies use different profiling approaches (V1V2 regions in ion torrent vs V3V4 regions in MiSeq), and they target broadly different populations with markedly different genetic backgrounds and lifestyles (adults in Japan vs adolescents in Spain), the similarities are striking. The two studies coincide in defining higher proportions of *Neisseria*, *Haemophilus*, and *Porphyromonas*, in one of the types (stomatotype 1, coinhabiting group 2), and those of *Prevotella*, and *Veillonella* in the other (stomatotype 2, coinhabiting group 1). That the two disparate studies agree in the two broadly defined groups strongly suggests that these two stomatotypes define two possible equilibria of oral microbial communities which are globally present. In addition, that the two stomatotypes are similarly identified in adult and adolescent datasets suggests that, despite important differences, oral microbiomes from these two age groups are similar at a broad level. This reinforces the idea that the two stomatotypes define global equilibria of microbial communities, despite a possibly large underlying diversity. We propose naming these stomatotypes *Neisseria-Haemophilus* (stomatotype 1) and *Prevotella-Veillonella* (stomatotype 2) based on the four most abundant genera among those driving their differences. Although other studies have defined higher number of clusters in the oral microbiome [16, 22], some of these clusters show clear similarities with the two stomatotypes found in this study.

We hypothesize that these two main stomatotypes are ubiquitous in human and that they can be found across geographical regions, ethnic groups, and lifestyles, pointing to inherently deep relationships between the human oral niches and the bacterial communities that colonize them. Further support of this hypothesis with broader studies in other populations and geographical regions is needed. This finding also opens the question of the stability of these two stomatotypes and how lifestyle may promote shifts between the two equilibria. It is unclear whether differences in the number of clusters found across studies are due to differences in the studied populations or to variations in the applied methods. In addition, some authors have warned about the necessity to consider variations among samples as a gradient rather than as discrete clusters [49]. We agree with this view and consider that stomatotypes represent trends in a continuous space of variation. As shown here, stomatotypes are appropriate to describe trends of change in the underlying microbial communities, which hint to shifts in the balance between driver genera. However, the two stomatotypes do contain a significant amount of variability and a gradient of variation, sometimes unrelated to the stomatotypes, is observed for the most abundant genera. In addition, that the described stomatotypes are common and globally distributed does not preclude the possibility that further, clearly distinct, stomatotypes may be found in other populations. Particularly, as the mentioned studies represent mostly healthy populations, further stomatotypes may be present that are associated to specific lifestyles or health conditions, which may represent alternative equilibria, or disbiotic alterations from the two described stomatotypes. Certainly, further studies including broader samples and specific sampling from different niches within the oral cavity will help us describe in more detail the oral microbial ecosystem and its interactions.

## Conclusions

The core oral microbiome described in this study is composed of genera commonly identified in other oral microbiome studies. We have shown that a number of diet and hygiene factors are associated with alterations in the composition of the oral microbiome, though one caveat is that, since the bulk of the sample set is from adolescents, some habits may be too recently developed to have already had a strong impact. The factor with the highest impact was the chemical composition of tap water from the hometowns of the donors. Indeed, most of the 17 ionic measurements showed significant correlations with a number of common genera such as *Veillonella* and *Porphyromonas*. This points to an important role of tap water in shaping the oral microbiome, which has been overlooked in previous studies.

We show that the samples can be clustered into two distinct groups which we call stomatotypes. The structures of these stomatotypes show notable similarities to the two clusters presented in another oral microbiome study of Japanese adults, despite differences in the technical approaches to the metagenomic analyses and highly distinct populations. Here, we propose the hypothesis that these two stomatotypes (the *Neisseria-Haemophilus* and *Prevotella-Veillonella* stomatotypes) represent global equilibria of oral microbial communities.

## Material and methods

### Sample collection

All participants, and at least one of their parents or legal guardians for those under the age of 18, signed a consent form to use their saliva samples for microbiome research. This consent form and the purpose of this project received approval by the ethics committee of the Barcelona Biomedical Research Park (PRBB). The target population was teenagers in the third course of Spanish secondary compulsory education (ESO), ages 13–15 years old. Additionally, we also collected samples from teachers of the participating schools. Schools were selected among those which volunteered to cover a broad range of Spanish provinces, a similar amount of schools in urban (towns or cities with more than 50,000 inhabitants) or rural (towns with less than 50,000 inhabitants and more than 50 km away from a large town) environments. Samples were collected during February to April in 2015. Participants were asked not to eat for 1 h prior to the sample collection. We tried to minimize variability as much as possible. To minimize sample collection variability, all donors received clear instructions on the procedure in person and sample collection was performed with the assistance of one researcher involved in the project, after a clear demonstration. All participants responded to a uniform questionnaire (see below). Before sample collection, saliva pH was measured using pH test strips (MColorpHast, Merck, range 5.0–10.0; 0.5 accuracy units). Although the use of pH test strips have been validated extensively [50], we validated our chosen strips. For this, we compared values given by eight different researchers using these strips to a scale of solutions with different pH to the values provided by a PHmeter (SevenEasypH model, Mettler-Toledo (GmbH). The correlation was high (*R*^2^ = 0.96), with average absolute differences between the value of the pH meter and that provided by the researcher being 0.33 which is within the range of the limit of detection of the method (0.5). Saliva samples were collected using a mouth wash and using a protocol that had been previously tested and compared with other procedures during a pilot phase of the project. Of note, this procedure is used in other oral microbiome studies and have been shown to produce consistent results with other sampling procedures [51]. The protocol used is as follows: Study participants rinsed their mouth with 15-mL sterile phosphate-buffered saline (PBS) for 1 min and subsequently returned the liquid into a 50-mL centrifuge plastic tube. The collected samples were centrifuged at 4500 g for 12 min at room temperature (r.t.) in an Eppendorf 5430 centrifuge equipped with an Eppendorf F-35-6-30 rotor. Pellets were resuspended with PBS, transferred to 1.5-ml eppendorf tubes and centrifuged at 4500 g for 5 min at r.t. using an Eppendorf FA-45-24-11-HS rotor. Supernatants were discarded, and pellets were frozen and kept at − 20 °C until the time of analysis.

### DNA extraction and sequencing

DNA from samples was extracted individually using the ZR-96 Fungal/Bacterial DNA kit (Zymo research Ref D6006) following manufacturer’s instructions. The extraction tubes were agitated twice in a 96-well plate using Tissue lyser II (Qiagen) at 30 Hz/s for 5 min at 4 °C. As a control for downstream procedures, we also used two DNA samples derived from bacterial mock communities obtained from the BEI Resources of the Human Microbiome Project: Each sample contained genomic DNA of ribosomal operons from 20 bacterial species. The HM-782D community contained an even number of ribosomal DNA per species (100,000 operons per species). The HM-783D community contained a variable number of operons, ranging from 1000 to 1000,000 per species.

DNA samples were diluted to 12.5 ng/μl and used to amplify the V3–V4 regions of 16S ribosomal RNA gene, using the following universal primers in a limited cycle PCR:

V3-V4-Forward

(5′-TCGTCGGCAGCGTCAGATGTGTATAAGAGACAGCCTACGGGNGGCWGCAG-3′)

V3-V4-Reverse

(5′-GTCTCGTGGGCTCGGAGATGTGTATAAGAGACAGGACTACHVGGGTATCTAATCC-3′)

The PCR was performed in 10-μl volume with 0.2-μM primer concentration. Cycling conditions were initial denaturation of 3 min at 95 °C followed by 20 cycles of 95 °C for 30 s, 55 °C for 30 s, and 72 °C for 30 s, ending with a final elongation step of 5 min at 72 °C. After this first PCR step, water was added to a total volume of 50 μl and reactions were purified using AMPure XP beads (Beckman Coulter) with a 0.9× ratio according to manufacturer’s instructions. PCR products were eluted from the magnetic beads with 32 μl of Buffer EB (Qiagen) and 30 μl of the eluate were transferred to a fresh 96-well plate.

The above described primers contain overhangs allowing the addition of full-length Nextera adapters with barcodes for multiplex sequencing in a second PCR step, resulting in sequencing ready libraries with approximately 450 bp insert sizes. To do so, 5 μl of the first amplification were used as template for the second PCR with Nextera XT v2 adaptor primers in a final volume of 50 μl using the same PCR mix and thermal profile as for the first PCR but only 8 cycles. After the second PCR, 25 μl of the final product was used for purification and normalization with SequalPrep normalization kit (Invitrogen), according to manufacturer’s protocol. Libraries were eluted in 20-μl volume and pooled for sequencing. Final pools were quantified by qPCR using Kapa library quantification kit for Illumina Platforms (Kapa Biosystems) on an ABI 7900HT real-time cycler (Applied Biosystems). Sequencing was performed in eight runs on an Illumina MiSeq with 2 × 300 bp reads using v3 chemistry with a loading concentration of 10 pM. In all cases, 15% of PhIX control libraries was spiked in to increase the diversity of the sequenced sample. Negative controls of the sample collection buffer, DNA extraction, and PCR amplification steps were routinely performed in parallel, using the same conditions and reagents. Our controls systematically provided no visible band or quantifiable DNA amounts by gel visualization or Bioanalyzer, whereas all of our samples provided clearly visible bands after 20 cycles. Four such controls were subjected to library preparation and sequenced. Expectedly, these sequenced non-template controls systematically yielded very few reads (a range of 30–880 reads per sample), in contrast to an average of 54,000 reads/library in sample-derived libraries.

### Pre-processing of 16S rRNA sequence reads and operational taxonomic unit assignment

The specific pipeline and parameters were set using sequence reads from both 16S rRNA amplicon and whole genome sequencing of the described mock communities. In the final adopted pipeline, reads were checked for quality using FastQC [52]. 16S amplicons were analyzed by Mothur v1.34.4 [53] following instructions described in the author’s website (https://www.mothur.org/wiki/MiSeq_SOP). Overlapping pairs of sequence reads were assembled, contigs with more than 4 ambiguities and shorter than 439 bp or larger than 466 bp were discarded, and the remaining contigs were aligned to the reference alignment provided by the SILVA database [54] (version 119) with a k-mer size of 8. Artifacts from the alignment and the contigs with more than 12 homo-polymers (the maximum number found in the reference alignment) were removed. The resulting alignment was simplified by removing the columns containing only gaps and by discarding duplicated sequences. The aligned sequences were then grouped allowing up to 4 mismatches and clusters with only one sequence were removed. Uchime (embedded in the Mothur framework) was used to remove chimeras, and the resulting sequences were classified according to the taxonomy into the corresponding operational taxonomic units (OTUs). Undesired lineages such as chloroplast, mitochondria, archaea, eukaryota, and “unknown” were removed. Sequences were then grouped again into OTUs by using the cluster.split command and considering the genus level. Finally, OTUs mapping to the same genus were grouped together.

### Microbiome composition profiling

The 16S rRNA OTU counts from the 1532 samples in this study for which we also had survey data were stored and analyzed using the R package Phyloseq (version 1.16.2) [55], which also has functions for filtering operational taxonomic units (OTUs), normalizing values, and various other calculations. One hundred eighty samples from 5 of the schools had to be removed due to an apparent batch effect during the sequencing procedure. This batch effect was detected in the initial quality assessment of the comparison of the data. In a diversity analysis these samples behaved very distinctly from the rest of the sample showing very low diversity values and corresponded to samples that had been processed and sequenced as a batch on the same day. Additionally, 33 samples were removed from the analyses because of errors with the sample identifiers, leaving a total of 1319 samples from 35 different schools from around Spain. Three hundred thirty-two different genera were identified in these samples. The 16S counts were normalized per sample, leaving the relative abundance of each genus within that sample, with all values between 0 and 100.

### Diversity measures

We estimated alpha diversity as measured by Shannon Diversity Index and Simpson Diversity Index [56] with the estimate_richness function from the Phyloseq package v1.16.2. We estimated beta diversity as the weighted and unweighted Unifrac distance between samples with the Unifrac function, as well as the Jensen-Shannon Divergence (JSD) with the JSD function, both from the Phyloseq package. In addition, we calculated the Bray-Curtis dissimilarity and Canberra index using the vegdist function in the vegan package (version 2.4.6) [57]. Both unifrac calculations require a phylogenetic tree which indicates phylogenetic distances by branch lengths. We obtained the tree by following the procedure described by Callahan et al. [58], wherein sequences are aligned, then using the R package phangorn (version 2.4.0), we construct a neighbor-joining tree and then fit a maximum likelihood tree. The weighted unifrac distance adds weights to the branch lengths based on relative abundance, while the unweighted unifrac distance considers only the presence or absence of OTUs. For each of these alpha and beta diversity measures, we also divided samples into quartiles in order to label each sample as having low (1st quartile), average (2nd and 3rd quartiles), or high diversity (4th quartile).

### Sample clustering

To cluster the samples in terms of their taxonomic composition (stomatotypes), we adapted the procedure described previously [31] for the determination of enterotypes, which we here refer to as stomatotypes. For this, we employed each of five beta diversity measures—Jensen-Shannon Divergence (JSD), weighted and unweighted UniFrac distance, Bray-Curtis dissimilarity, and Canberra index—to produce distance matrices for the genera of all samples and then Partitioning Around Medoids (PAM) clustering to group samples with similar overall oral microbiomes. Next, we used the Calinski-Harabasz (CH) index [59] to determine the optimal number of clusters, and we further verified this by calculating the average silhouette width of the samples, which is a measure of the separation of samples within one cluster from those of another cluster, as well as the prediction strength, another measure of the efficiency of clustering. The functions for these calculations come from the R packages cluster v2.0.6 (https://cran.r-project.org/package=cluster), clusterSim v0.45-1 (https://cran.r-project.org/package=clusterSim), and fpc v2.1-11.1 (https://CRAN.R-project. =org/package=fpc). Clustering was validated using all five distance measures to ensure proper clustering, but analyses here are performed using the clustering based on JSD. As detailed in Bork’s group tutorial (http://enterotype.embl.de/enterotypes.html), we used the R package ade4 v1.7-4 (https://cran.r-project.org/package=ade4) for visualization. We first excluded those genera that are potentially noisy, removing those for which the average relative abundance across all samples was lower than 0.01%. We then used Between Class Analysis (BCA) to determine the “drivers” for each stomatotype, which are the genera accounting for the greatest separation between samples of a given stomatotype from the other types. We used a Principal Coordinate Analysis (PCoA) to visualize the clustering of the samples within their respective stomatotypes. Furthemore, the adonis function in the vegan package was used to perform a PERMANOVA test on each beta diversity measure to ensure significant separation of stomatotypes.

### Gradients of abundances

The gradients of abundances were displayed using the same coordinates in the PCoA plots described above, and points were colored based on abundances of the indicated taxa binned into every 10th percentile of those abundances. Shapes of points are determined by the stomatotype based on a given distance measure, typically the JSD measure in figures here.

### Co-occurrence networks

To produce co-occurrence networks of genera within a given stomatotype, we use the R packages sna v2.4 (https://cran.r-project.org/package=sna) and network v1.13.0 (https://cran.r-project.org/package=network). We first calculated Pearson correlations between pairs of genera within samples of a given stomatotype and used the Bonferroni correction to adjust the *p* values. Then, considering the 20 most common genera within the samples of a given stomatotype, we produce a network wherein edges are formed between only those genera that have a correlation coefficient greater than 0.25 or less than − 0.25 and an adjusted *p* value less than 0.05. Red edges indicate positive correlations, blue edges indicate negative correlations and edge width is proportional to the absolute value of the correlation coefficient. Vertex color is based on the phylum to which the given genus belongs.

### Questionnaire and other metadata

Participants were asked to answer one questionnaire inquiring about aspects relevant to their hygiene and dietary habits. These questions were adapted from questionnaires available at the PhenX toolkit (consensus measures for Phenotypes and eXposures), which provides recommended standard data collection protocols for conducting biomedical research [60] and which has been recommended by the microbiome research community [61]. In addition, some of the questions were selected among those suggested by citizens themselves through the project’s website. The final questionnaire is available at (Additional file 2). Data on average socioeconomic status of each participant high school was obtained as follows. We first assigned geographic coordinates to all schools based on their postal address, which were used to assign socioeconomic values from their districts using the GIS (Geographic Information System) software QGIS v.2.14 and based on the Census Tracts of 2001, of the Urban Vulnerability Atlas Database from the Spanish government (http://www.fomento.gob.es/MFOM/LANG_CASTELLANO/DIRECCIONES_GENERALES/ARQ_VIVIENDA/SUELO_Y_POLITICAS/OBSERVATORIO/Atlas_Vulnerabilidad_Urbana/). Data on tap water hardness was obtained from several national ionic composition studies [40–42].

### Statistical analyses

We obtained the Pearson correlation coefficient between abundances of pairs of genera, between genera and other continuous variables (i.e., questionnaire answers, pH), and between pairs of variables. We performed the Kruskal-Wallis rank sum test between categorical variables (i.e., questionnaire, stomatotype) and abundances or other continuous variables. In those cases where the Kruskal-Wallis test was statistically significant, the differential groups and the direction of their difference (greater or less than other groups) was determined by ANOVA using the aov and TukeyHSD functions from the base R package stats v3.4.1. We also performed chi-squared tests between categorical values as well as between those variables and the presence/absence of OTUs. In all cases, we applied the Bonferroni correction to adjust the *p* values by the number of comparisons. Correlation heatmaps, boxplots, and volcano plots were generated using ggplot2 v2.2.1 (https://cran.r-project.org/package=ggplot2), and association plots were generated using the assoc function from the R package vcd v1.4-3 (https://cran.r-project.org/package=vcd). In general, all of our statistical analyses considered all 1319 samples, except for the instances that are specifically mentioned in the text (i.e., by referring to a correlation affecting students), we did so with subsets of the samples, including students only (1297 of the 1319 samples) or those samples not drinking primarily from bottled water (814 of the 1319 samples). To assess the robustness of correlations with pH to stochastic variations within the precision range of the measurements, we performed a computational test, changing measured pH value of each saliva sample to a random number within the precision range (± 0.5). We repeated this 1000 times and measured whether reported significant correlations were still existing. For all reported correlations, they remained in 100% of the cases.

### Distribution maps

We produced maps with distributions of various values using shape files for Spain obtained from the GADM database of Global Administrative Areas (http://gadm.org/). We used the readShapeSpatial function from the R package maptools v0.9-2 (https://cran.r-project.org/package=maptools) which creates a Spatial DataFrame object that can be used to plot values in different regions of a map, and the boxed.labels function from the R package plotrix v3.6-6 (https://cran.r-project.org/package=plotrix) to include labels for regions on the figure.

## Additional files

Additional file 1: **Figure S1.** Sample collection sites. Samples were collected from 40 different schools in 30 cities across Spain. This figure shows the locations of the cities from which the samples were collected with the corresponding names listed on the left and the number of samples next to it. Region names are shown in the map. (PDF 226 kb)

Additional file 2: **Table S1.** Questionnaire and metadata: Table of question/variable IDs found in the other supplementary tables along with descriptions of their values. Some questions have multiple columns as we explored the question from multiple angles (i.e., yes/no, frequencies, ranges of values in order to account for outliers, or grouping by quartiles). (XLS 16 kb)

Additional file 3: **Figure S2.** Distributions of diversity values across all samples. (a) Shannon alpha diversity. (b) Weighted UniFrac distances (beta diversity). (PDF 74 kb)

Additional file 4: **Table S2.** Genus vs genus correlations: Table of Pearson correlations between the relative abundances of genera among all samples, includes only those genera present in at least 20 samples. Values shown are Pearson correlation coefficients in those cases where Bonferroni-adjusted *p* values were less than 0.05. Figures 2 and 4 show only the 67 genera which were present in at least 1/3 of all samples (436) for the sake of visual clarity, but this table contains values for all 141 genera that were present in at least 20 samples. The order of rows and columns correspond to those presented in Fig. 2a and Fig. 5. (XLS 77 kb)

Additional file 5: **Table S3.** Kruskal-Wallis significant differences: Table of Bonferroni-adjusted *p* values from Kruskal-Wallis rank sum tests between relative abundances of genera, questionnaire responses and other continuous metadata variables with all categorical questionnaire responses and metadata variables. Values shown are the adjusted *p* values that are less than 0.05, indicating that at least one group in the categorical variable has a significantly different mean value than other groups for the continuous variable. Only contains rows and columns that have at least one such significant *p* value. In the case of significant *p* values, groups and their directionality were then determined by ANOVA. (XLS 38 kb)

Additional file 6: **Table S4.** Chi-squared tests: Table of Bonferonni-adjusted *p* values from chi-squared between categorical questionnaire responses, other continuous metadata variables, and the presence/absence of the genera detected in this study. Values shown are the adjusted *p* values that are less than 0.05, indicating that there is an association between at least one group of each variable. This table only contains rows and columns that have at least one such significant *p* value. In the case of significant *p* values, specific associations were determined by producing association plots as described in the “Materials and methods” section. (XLS 125 kb)

Additional file 7: **Table S5.** Genus vs question correlations: Table of Pearson correlations between the relative abundances of genera and questionnaire responses with continuous values and other metadata variables, includes only those genera present in at least 20 samples. Values shown are Pearson correlation coefficients in those cases where Bonferroni-adjusted *p* values were less than 0.05. Only contains rows and columns that have at least one such significant *p* value. (XLS 31 kb)

Additional file 8: **Table S6.** Question vs question correlations: Table of Pearson correlations between all questionnaire responses with continuous values and other metadata variables among all samples. Values shown are Pearson correlation coefficients in those cases where Bonferroni-adjusted *p* values were less than 0.05. Only contains rows and columns that have at least one such significant *p* value. (XLS 25 kb)

Additional file 9: **Figure S3.** Distribution of oral pH. Histogram of the pH of donors’ saliva prior to sample collection. (PDF 46 kb)

Additional file 10: **Table S7.** Genus/question vs water value correlations: Table of Pearson correlations between the relative abundances of genera, questionnaire responses, and other metadata variables with measurements of various components of tap water. Samples that primarily drank from bottled water (505 out of 1319) at home were excluded here. Values shown are Pearson correlation coefficients in those cases where Bonferroni-adjusted *p* values were less than 0.05. Only contains rows and columns that have at least one such significant *p* value. (XLS 9 kb)
