# Supplementary figures and images for: Citizen science charts two major “stomatotypes” in the oral microbiome of adolescents and reveals links with habits and drinking water composition
Source: Microbiome. 2018 Dec 6;6:218. doi: 10.1186/s40168-018-0592-3 (PMC6284318; doi:10.1186/s40168-018-0592-3)

**Supplementary Figure S1: Sample collection sites**

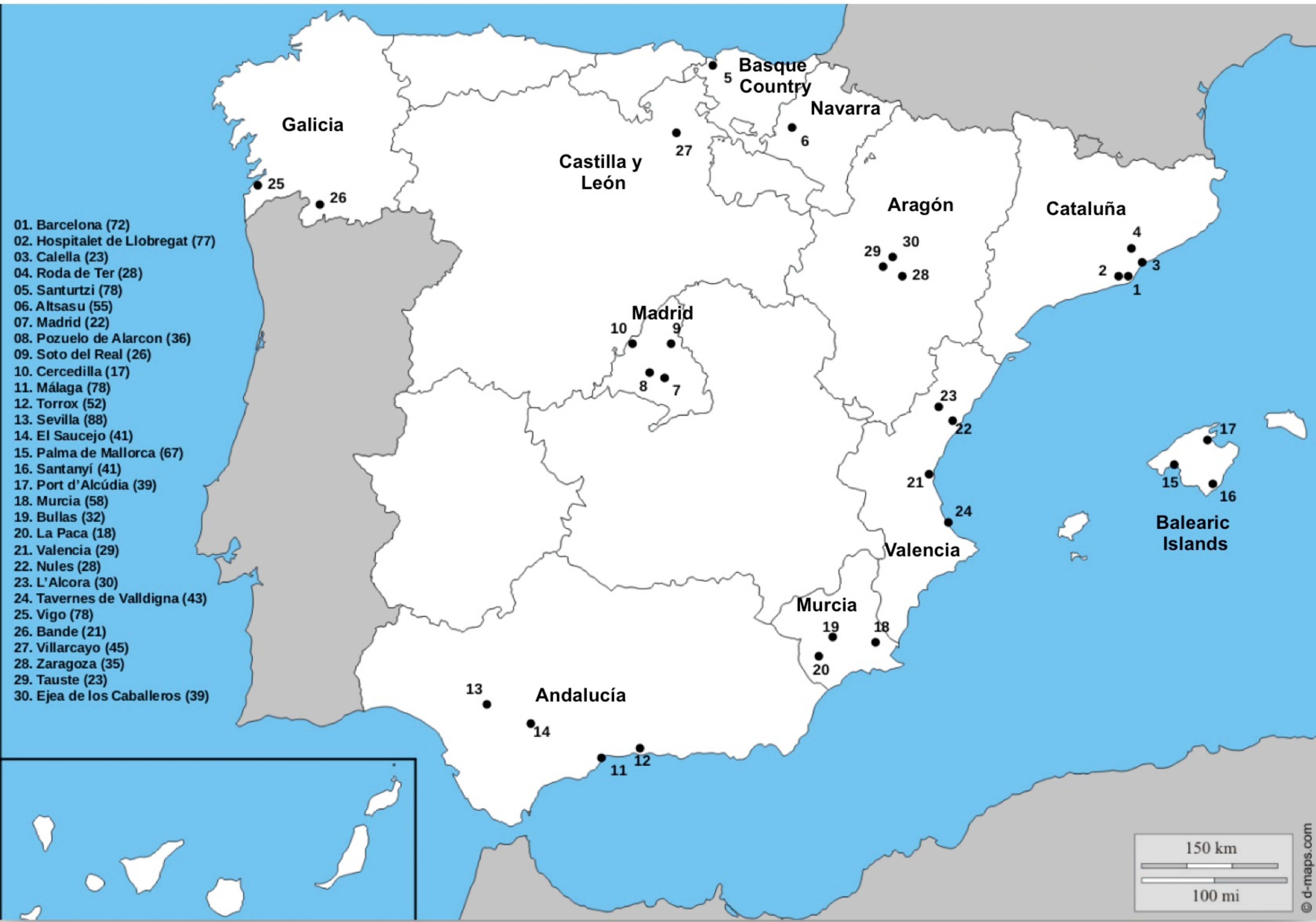

Supplement: Supplementary file 1 — Figure S1. Sample collection sites. Samples were collected from 40 different schools in 30 cities across Spain. This figure shows the locations of the cities from which the samples were collected with the corresponding names listed on the left and the number of samples next to it. Region names are shown in the map. (PDF 226 kb) [file 40168_2018_592_MOESM1_ESM.pdf]

## Supplementary Figure S2: Distributions of diversity values across all samples

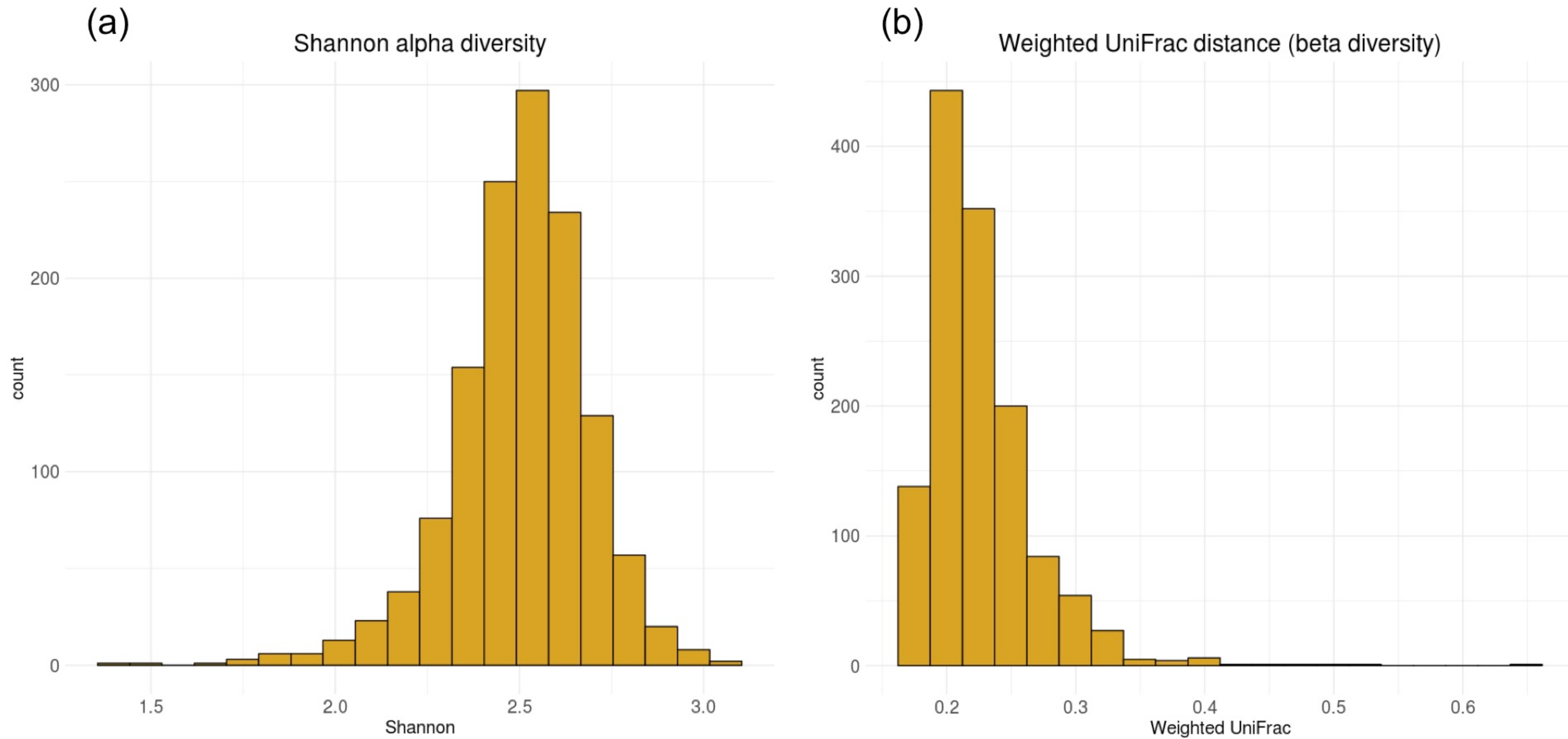

Supplement: Supplementary file 3 — Figure S2. Distributions of diversity values across all samples. (a) Shannon alpha diversity. (b) Weighted UniFrac distances (beta diversity). (PDF 74 kb) [file 40168_2018_592_MOESM3_ESM.pdf]

**Supplementary Figure S3: Distribution of oral pH**

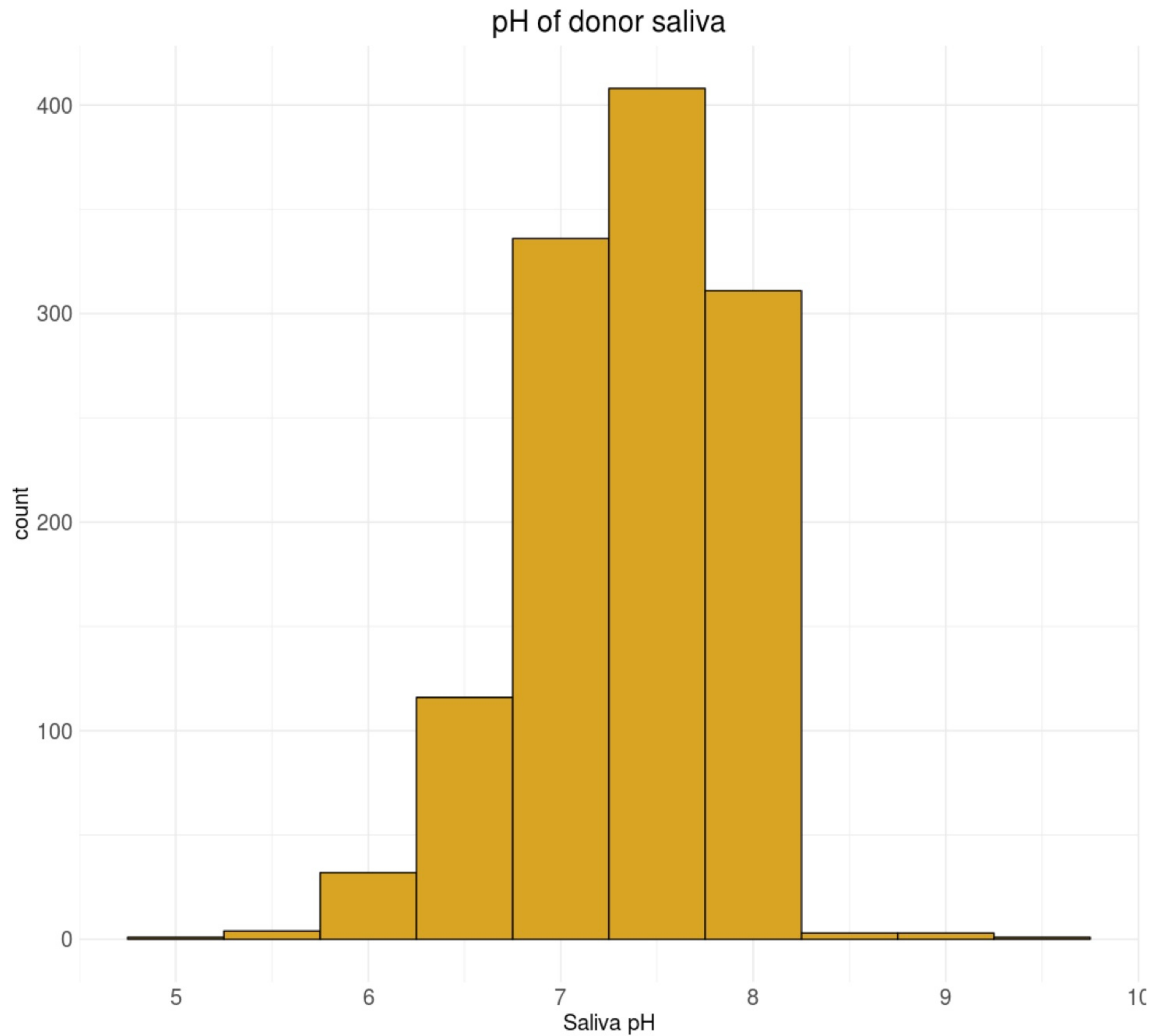

Supplement: Supplementary file 9 — Figure S3. Distribution of oral pH. Histogram of the pH of donors’ saliva prior to sample collection. (PDF 46 kb) [file 40168_2018_592_MOESM9_ESM.pdf]
